# Emergency reversal of Rivaroxaban with Andexanet alfa in a child with hemorrhagic brain metastasis from Wilms tumor

**DOI:** 10.1007/s00381-026-07286-w

**Published:** 2026-05-02

**Authors:** Artem Rafaelian, Sae-Yeon Won, Luisa Mueller, Moritz Armbrust, Katharina J. Weber, Annika Haas, Daniel A. Reuter, Thomas M. Freiman, Florian Gessler, Christian Spang, Almut Meyer-Bahlburg, Peter Hingst, Carl Friedrich Classen, Daniel Dubinski, Teresa Marta Cardesa-Salzmann

**Affiliations:** 1 Department of Neurosurgery, Rostock University Medical Center, Rostock, Germany; 2 Goethe University, University Hospital Frankfurt, Neurological Institute (Edinger Institute), Frankfurt am Main, Germany; 3 Department of Anesthesiology, Intensive Care Medicine and Pain Therapy, Rostock University Medical Centre, Rostock, Germany; 4 Department of Pediatrics and Adolescent Medicine, Rostock University Medical Center, Rostock, Germany; 5 Department of Pediatrics and Adolescent Medicine, Section for Pediatric Hematology & Oncology and Palliative Care, Rostock University Medical Center, Rostock, Germany; 6 German Cancer Consortium (DKTK), Partner site Frankfurt / Mainz and German Cancer Research Center (DKFZ), Heidelberg, Germany; 7 Goethe University, University Hospital Frankfurt, Frankfurt Cancer Institute (FCI), Frankfurt am Main, Germany; 8 Goethe University, University Hospital Frankfurt, University Cancer Center (UCT) Frankfurt-Marburg, Frankfurt am Main, Germany

**Keywords:** Rivaroxaban, DOAC, Andexanet alfa, Pediatric anticoagulation, Intracranial hemorrhage

## Abstract

**Background:**

Central nervous system (CNS) metastases from Wilms tumor (WT) are exceedingly rare. Intracerebral hemorrhage secondary to metastatic WT is even less common, and the management of such cases is further complicated when patients are receiving a direct oral anticoagulant (DOAC) like Rivaroxaban, for which pediatric reversal guidelines are lacking.

**Case presentation:**

We report on the case of a 5-year-old boy with relapsed stage IV Wilms tumor who presented with rapidly progressive neurological deterioration caused by brain metastases with extensive intraparenchymal and intraventricular hemorrhage while receiving Rivaroxaban due to prior thrombosis. An emergent craniotomy and tumor resection was safely performed after emergent reversal of anticoagulation with Rivaroxaban using Andexanet alfa, administered in this pediatric patient with off-label consent in the setting of a life-threatening intracranial hemorrhage requiring emergent neurosurgical intervention. No excessive intraoperative bleeding was noted. Treatment for relapsed WT according to the SIOP-UMBRELLA-Protocol was initiated. Three weeks after Andexanet alfa treatment, a thrombotic event in the left iliac veins occurred, requiring anticoagulation with unfractionated heparin.

**Conclusions:**

This case highlights the therapeutic challenges of managing intracranial hemorrhage in a pediatric patient requiring emergent neurosurgical debulking in the setting of Rivaroxaban anticoagulation. To our knowledge, this is the second case reporting on Rivaroxaban reversal through Andexanet alfa in children. Early multidisciplinary intervention, meticulous neurosurgical management and continuation of oncologic therapy can lead to favorable outcomes even in such complex presentations.

## Introduction

Wilms tumor (WT) is the most frequent kidney tumor in infants and children [1]. The mean age at diagnosis is 44 months in unilateral cases and 31 months in bilateral cases, with about 10% of children with WT having an associated congenital malformation syndrome [2, 3]. Approximately 10% of patients with WT present with hematogenous metastases, most frequently to the lungs (85%), followed by the liver (10%), and only very rarely to the bones or brain [2]. Tumor recurrence rates are 15% for favorable histology and 50% for anaplastic histology WT [4]. The most common sites of relapse are the lungs, followed by the abdomen, flank and liver. Overall, cerebral (0.5%) or bone metastases are rare in children with WT [5].

Rivaroxaban is a direct factor Xa inhibitor belonging to the group of the direct oral anticoagulants (DOACs), which has demonstrated favorable pharmacokinetic and safety profiles in recent trials, supporting its increasing use in children, including pediatric oncology patients due to its efficacy, safety and ease of use [6, 7]. However, data on the management of major bleeding events in pediatric patients receiving DOACs remain very limited [8]. Andexanet alfa, a recombinant, catalytically inactive modified factor Xa, is currently the only approved agent for the reversal of Rivaroxaban and Apixaban in adults with life-threatening bleeding [9]. Data on dosing and efficacy of Andexanet alfa are lacking in the pediatric age group.

Importantly, the available evidence on andexanet alfa is largely derived from non-surgical adult populations, and its use in emergency neurosurgical settings remains insufficiently characterized. Emerging data suggest that perioperative administration may be associated with a distinct safety profile, particularly with regard to thromboembolic complications [10].

Herein we report on the case of a pediatric patient with relapsed WT with multiple bilateral brain metastases complicated by intracerebral hemorrhage while on Rivaroxaban anticoagulation, requiring emergent craniotomy and anticoagulation reversal with Andexanet alfa.

## Case presentation

**Fig. 1:**
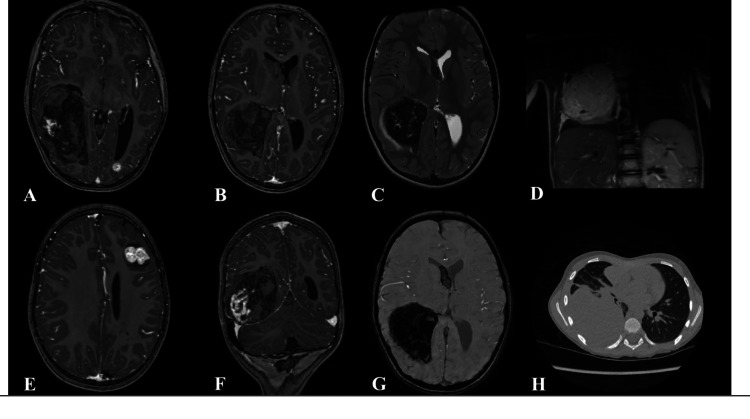
Magnetic resonance imaging (MRI) of the brain and computed tomography (CT) of the thorax demonstrating multiple supratentorial metastatic lesions with extensive intratumoral and intraventricular hemorrhage. **A, B, E** Axial T1-weighted images with contrast enhancement and** F** coronal T1-weighted image with contrast enhancement show multiple enhancing metastatic masses with hemorrhagic components. **C** Axial T2 FSE sequence and **G** SWI sequence visualize the intraventricular blood products and surrounding oedema. **D** Coronal T2-weighted image of the thorax image and **H** axial CT-thorax images showing large multilobed right-sided pulmonary mass

A 5-year-old male patient with a past medical history of stage IV WT with pulmonary metastases was admitted to the pediatric oncology ward with dry cough and chest pain. A chest computed tomography (CT) showed a large multi-lobar right-sided pulmonary mass, suspicious of metastasis (Fig. 1).

Nine months prior he had completed first line therapy for stage IV WT within the UMBRELLA-SIOP-RTSG-Protocol. He had received 6 weeks preoperative chemotherapy with Actinomycin D, Vincristine and Doxorubicin (AVD), followed by tumor nephrectomy, flank radiotherapy (14.4 Gy) for local stage III disease and adjuvant chemotherapy with AVD250. Tumor nephrectomy had shown a nephroblastoma with nearly completely regressive changes, with a tumor thrombus at the resection margins of the renal vein. Surgical sampling of one of the persistent pulmonary nodules showed complete necrosis; therefore, lung irradiation was deemed unnecessary. After vena cava endoplasty in the setting of tumor nephrectomy with the presence of a tumor thrombus in the vena cava inferior he developed a mural thrombosis in the vena cava inferior. Therapeutic Enoxaparin anticoagulation was started and was later switched to Rivaroxaban treatment (5 mg per os (p.o) every 12 h) for convenience. Rivaroxaban-anti-Xa levels had been monitored and were within therapeutic range (53.3 ng/ml (20–500 ng/mL)).

Shortly after admission the patient developed headaches, vomiting, followed by altered mental status. He was transferred to the intensive care unit. An emergency MRI of the brain demonstrated multifocal cerebral metastases with three metastases with hemorrhagic transformation. The largest hemorrhagic lesion in the right parieto-frontal region measured 83 × 48 × 56 mm, with intralesional and intraventricular hemorrhage as well as a 9-mm midline shift (Fig. 1). Quick and partial thromboplastin time (PTT) were within normal limits (85%, 28.6 s). The complete blood count showed normal thrombocyte values (230,000/mm^3^) and creatinine was within normal limits (22.5 µmol/L). The patient had received Rivaroxaban 5 mg p.o 12 h prior to the event. His weight was 17 kg.

**Fig. 2:**
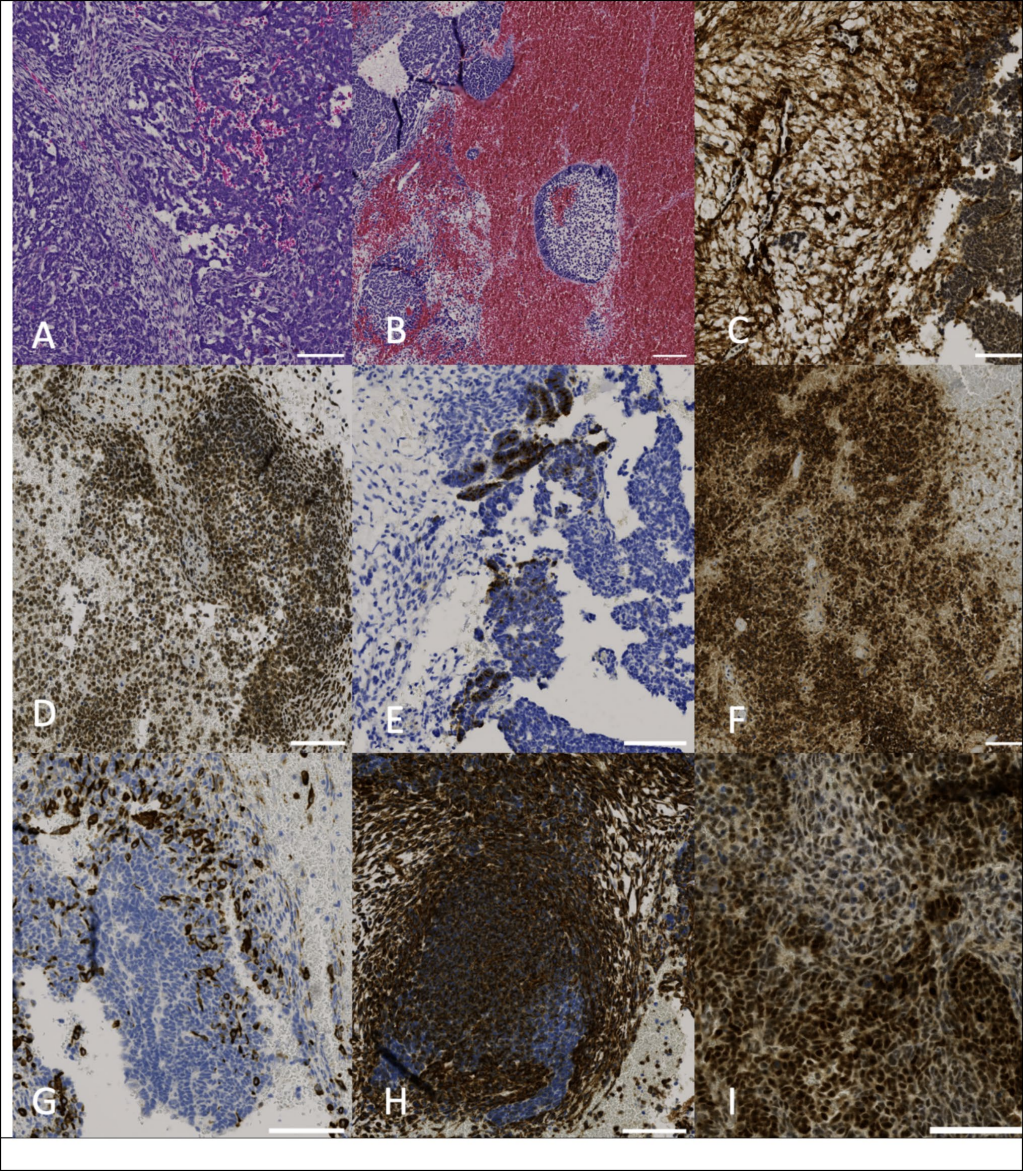
Tumor histology and immunohistochemistry. Scales bars indicate 100 µm, respectively. **A**, **B** H&E-Stain: Highly cellular small-round-blue-cell tumor with stromal and blastemal components and massive hemorrhage. **C** The tumor demonstrated SMA immunoreactivity especially within the stromal component. **D** Nuclear INI-1 expression was preserved within tumor cells. **E**, **F**, **G**, **H**, **I** EMA, CD56, Desmin, Vimentin and nuclear PAX 8 immunoreactivity within the tumor. The tumor was negative for panCK, S100 and GFAP (not shown)

Reversal of Rivaroxaban in the setting of active bleeding and the need for immediate neurosurgical intervention was mandatory. Off-label consent for Andexanet alfa use was obtained. Andexanet alfa was administered first 100 mg as an intravenous (IV) bolus over 15 min followed by 100 mg IV infusion over 100 min as described in the only published pediatric case report on the use of Andexanet alfa in a pediatric patient with similar weight [11]. Rivaroxaban anti-Xa levels after Andexanet alfa, prior to start of surgery, were 2.1 ng/ml (20–500 ng/mL). During transport to the operating theatre, the patient’s Glasgow Coma Scale score declined to 6 and anisocoria developed, with the right pupil larger than the left. An emergency craniotomy with tumor resection, intraoperative neurophysiological monitoring, and placement of an external ventricular drain was performed without excessive intraoperative bleeding. The duration of surgery was 124 min. No intraoperative transfusion of red blood cells or coagulation products was required. Hemoglobin levels showed a moderate decrease from 9.67 g/dl preoperatively to 8.38 g/dl immediately after surgery. The day after surgery his hemoglobin had dropped to 6.12 g/dl. He was transfused with one unit of irradiated packed red blood cells after which his hemoglobin levels remained stable. The patient was extubated the same evening, exhibiting left-sided hemiparesis and facial paresis. The postoperative MRI showed complete resection of the hemorrhagic metastasis without signs of a rebleeding. Histopathology confirmed metastatic WT to the brain (Fig. 2).

**Fig. 3:**
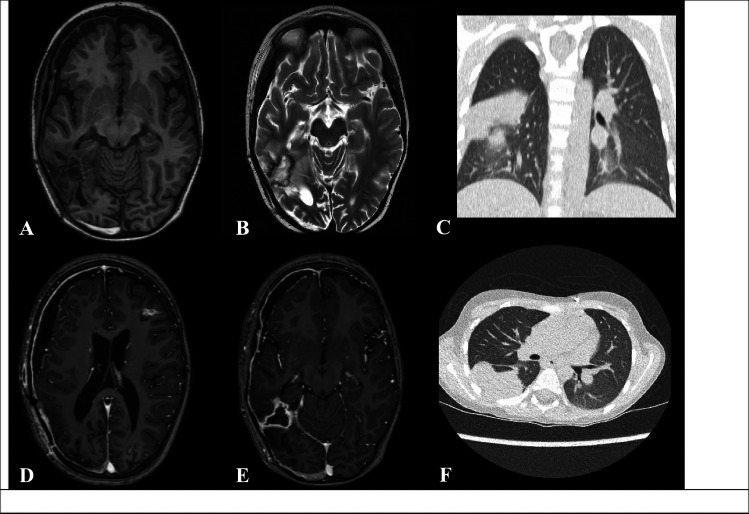
Magnetic resonance imaging of the head and computed tomography scan after two chemotherapy cycles. Axial images demonstrating very good partial response with expected postoperative changes. **A** T1-weighted image.** B** T2-weighted image showing oedema resolution post tumor resection. **D** Contrast-enhanced T1-weighted image. **E** Contrast-enhanced T1-weighted image demonstrating interval reduction in the size of a separate metastatic lesion under ongoing chemotherapy, indicating a favorable treatment response. **C** coronal and **F** axial CT-thorax images

Resumption of anticoagulation was omitted in the postoperative period and during the following weeks due to a high estimated risk of repeat intracranial bleeding of the remaining cerebral metastases. Mechanical thromboprophylaxis was applied during this period. A Hickman catheter was placed, and chemotherapy according to the UMBRELLA relapse protocol group BB was initiated with alternating cycles of Ifosfamide, Carboplatin, and Etoposide (ICE) and Cyclophosphamide, Carboplatin, and Etoposide (CCE) every 21 days. On postoperative day 21, the patient developed diffuse swelling of the left lower limb. An MRI of the pelvis revealed a deep venous thrombosis involving the left iliac veins. Unfractionated heparin was started to maintain PTT 60–80 s. No bleeding events occurred. After 2 weeks, the patient was transitioned to enoxaparin 1 mg/kg every 12 h subcutaneously to maintain anti-Xa levels in a therapeutic range (0.5–0.8 IU/mL). In a follow-up Doppler ultrasound 2 months later, the thrombosis in the left iliac veins had completely resolved. Restaging imaging procedures prior to the third chemotherapy cycle showed a very good partial response both in the CNS and in the right lung (Fig. 3). From the neurologic standpoint, gradual motor improvement occurred over several weeks with physiotherapy, assisted standing, orthotic support, and mobility aids. At discharge, the modified Rankin Scale score was 3.

## Discussion

Metastatic spread to the CNS and CNS recurrences in WT are an extremely rare event [12–14]. Even more infrequent is the presence of intralesional bleeding in WT CNS metastasis. Patients with WT recurrence involving the brain may have durable survival with multimodality therapy [15, 16]. Therefore, in many instances, a curative multidisciplinary therapeutic approach is justified.

In this case, brain metastasis with extensive intraparenchymal and intraventricular hemorrhage while receiving Rivaroxaban after vena cava inferior endoplasty and mural thrombosis posed a special challenge to the emergency multidisciplinary treatment.

The main risk of anticoagulation is bleeding, and intracranial hemorrhage (ICH) is the most feared manifestation. Rivaroxaban is a direct factor Xa inhibitor widely used both in adult and pediatric patients due to its efficacy, safety, and convenience by oral dosing, with U.S. Food and Drug Administration (FDA) and the European Medicines Agency (EMA) approval for its use in pediatric patients [17]. There were no major bleeding events in the rivaroxaban phase 3 pediatric clinical trial and the rate of clinically relevant non-major bleeding was 3% [18]. Published data related to the use of DOACs in pediatric cancer patients report on no recurrent thrombosis and no major bleeding events, with promising results for Rivaroxaban use in children with cancer [6, 7, 19]. Despite the lack of major bleeding events in the Rivaroxaban trials, emergency reversal of DOACs in the event of major bleeding poses a great challenge for children because pediatric clinical guidelines for DOAC reversal are lacking.

Importantly, the available evidence on andexanet alfa is largely derived from non-surgical adult populations included in pivotal clinical trials [20]. In contrast, our case represents an emergency neurosurgical scenario, in which the safety and efficacy of andexanet alfa remain insufficiently characterized. Emerging data suggest that perioperative use of andexanet alfa may be associated with a distinct safety profile, particularly with regard to thromboembolic complications [10].

Andexanet alfa is approved by the US Food and Drugs Administration and the European Medicines Agency (EMA) for adults for reversal of rivaroxaban and apixaban, but data on dosing and efficacy of Andexanet alfa are lacking in the pediatric age group [21]. Andexanet alfa is a recombinant, catalytically-inert factor Xa variant that has higher binding affinity for direct factor Xa inhibitors than does natural factor Xa, developed for rapid and effective reversal of factor Xa inhibitor-induced anticoagulation within minutes [22]. Two phase 2, randomized, double-blind, placebo-controlled studies have shown efficacy in reversal and safety in adults [9, 20]. Restoration of hemostasis after Andexanet alfa was judged to be good or excellent in 80% of Rivaroxaban-treated patients. At 30 days, a thrombotic event occurred in 10% of patients [22]. Importantly, the available evidence on Andexanet alfa is largely derived from non-surgical adult populations included in pivotal clinical trials [20]. Emerging data suggest that perioperative use of Andexanet alfa in adults may be associated with a distinct safety profile, particularly with regard to ischemic complications [10]. In adults, in settings where Andexanet alfa is not available, non-specific pro-hemostatic agents such as four-factor prothrombin complex concentrates (4F-PCC) are commonly used as an off-label, non-specific factor replacement approach to manage Rivaroxaban-associated life-threatening bleeding when Andexanet alfa is not available [23–26]. Efficacy and safety data regarding prothrombin complex concentrates for children with DOAC-associated bleeding are lacking [27]. Studies in adults point towards a superiority of Andexanet alfa to 4F-PCC for the treatment of Rivaroxaban-associated intracranial bleeding events [28]. However, there is a lack of evidence regarding the use of Andexanet alfa and prothrombin complex concentrates for anticoagulation reversal of direct factor Xa inhibitors in the pediatric population. To date, only one pediatric case report by Takasaki et al. [11], described successful reversal of Rivaroxaban with Andexanet alfa in a child. In this case, given the presence of life-threatening intracranial hemorrhage, signs of impending herniation, and the need for immediate neurosurgical intervention, we opted for reversal with Andexanet alfa, based on its specific mechanism of action, its potential for rapid neutralization of the anticoagulant effect of Rivaroxaban and the publication by Takasaki et al supporting its efficacy and safety in a pediatric patient of similar weight requiring emergent neurosurgical intervention. While both cases describe successful reversal of Rivaroxaban with Andexanet alfa in pediatric patients, the case described by Takasaki [11] presented with a subdural hematoma requiring emergent burr hole hematoma evacuation, whereas in this case, the patient had cerebral metastases with extensive intratumoral and intraventricular hemorrhage and signs of intracranial hypertension, with the need for immediate hematoma evacuation, tumor resection, intraoperative neurophysiological monitoring, and placement of an external ventricular drain. This highlights the additional complexity of operative management of this case and further underscores the clinical relevance of our report. 

Anti-Xa-levels for Rivaroxaban show modest value for prediction of bleeding events in adults [29]. We did not collect immediate pre-reversal anti-Xa levels for Rivaroxaban in the emergency situation of the intracranial bleed. Months prior, anti-Xa levels for rivaroxaban had been within normal range (53.3 ng/ml (20–500 ng/mL)) and the patient remained on a stable dosing schedule of rivaroxaban of 5 mg p.o twice daily. In this case, anti-Xa activity after Andexanet alfa treatment prior to emergency surgery was evaluated, showing very low anti-Xa activity levels for rivaroxaban (2.1 ng/m; prior measured level 53.3 ng/ml (20–500 ng/mL)). Intraoperatively, no excessive bleeding was noted.

In this patient, a thrombotic event in the left iliac veins occurred three weeks after administration of Andexanet alfa. However, this finding must be interpreted with caution. The patient had multiple predisposing risk factors for thrombosis, including active malignancy, a history of prior thrombosis, reduced mobility, and ongoing chemotherapy. Therefore, a causal relationship between Andexanet alfa and the thrombotic event cannot be definitively established, and the association is likely multifactorial. Although Andexanet alfa has been associated with thrombotic events in adult studies, this observation in our case should be interpreted within the broader clinical context [9]. 

In summary, there is an urgent need to establish pediatric clinical guidelines for DOAC reversal and research is needed to determine the dosing, efficacy, and safety of reversal agents as well as to identify risk factors for bleeding with DOACs. Evidence for Andexanet alfa in children after Rivaroxaban exposure is sparse and safety data-particularly regarding thrombotic risk-are insufficient. This case highlights the exceptional rarity of CNS metastases in WT and demonstrates real-world decision-making regarding DOAC reversal in a high-risk pediatric oncology patient, adding to the limited literature on Andexanet alfa use in children.

## Conclusion

This case underscores the importance of rapid multidisciplinary decision-making in managing intracerebral hemorrhage in pediatric patients receiving DOAC therapy. Although Andexanet alfa is not approved for use in children, its administration in life-threatening situations where urgent reversal of factor Xa inhibition is required may be lifesaving as described in this case. Our experience also demonstrates that active, intensive, combined treatment, including emergent surgical treatment and timely initiation of chemotherapy, can lead to favorable clinical outcomes. Further research and the development of pediatric-specific guidelines are needed to optimize the management of severe bleeding events and anticoagulation reversal in this vulnerable population.

## Data Availability

No datasets were generated or analyzed during the current study.
